# Supplementary material for: Classification of Sleep Quality and Aging as a Function of Brain Complexity: A Multiband Non-Linear EEG Analysis
Source: Sensors (Basel). 2024 Apr 28;24(9):2811. doi: 10.3390/s24092811 (PMC11086092; doi:10.3390/s24092811)
Supplement: Supplementary file 1 [file sensors-24-02811-s001.zip › sensors-2950325-supplementary.pdf]

## Supplementary Material

## Accuracies reached using different machine learning methods

Young adults with good sleep quality vs older adults with bad sleep quality:

| FineTree | | | | | |
|---|---|---|---|---|---|
| Channel | Gamma | Beta | alpha | Theta | Delta |
| Mean | 63% | 67% | 73% | 77% | 78% |
| Max | 83% | 89% | 86% | 89% | 92% |

| MediumTree | | | | | |
|---|---|---|---|---|---|
| Channel | Gamma | Beta | alpha | Theta | Delta |
| Mean | 63% | 67% | 73% | 77% | 78% |
| Max | 83% | 89% | 86% | 89% | 92% |

| CoarseTree | | | | | |
|---|---|---|---|---|---|
| Channel | Gamma | Beta | alpha | Theta | Delta |
| Mean | 63% | 67% | 73% | 77% | 78% |
| Max | 83% | 89% | 86% | 89% | 92% |

| LogisticRegression | | | | | |
|---|---|---|---|---|---|
| Channel | Gamma | Beta | alpha | Theta | Delta |
| Mean | 62% | 66% | 72% | 72% | 75% |
| Max | 81% | 81% | 89% | 89% | 97% |

| MediumGuassianSVM | | | | | |
|---|---|---|---|---|---|
| Channel | Gamma | Beta | alpha | Theta | Delta |
| Mean | 69% | 69% | 77% | 79% | 86% |
| Max | 78% | 83% | 86% | 89% | 92% |

| CoarseGuassianSVM | | | | | |
|---|---|---|---|---|---|
| Channel | Gamma | Beta | alpha | Theta | Delta |
| Mean | 69% | 69% | 69% | 69% | 69% |
| Max | 69% | 69% | 69% | 69% | 69% |

| FineKNN | | | | | |
|---|---|---|---|---|---|
| Channel | Gamma | Beta | alpha | Theta | Delta |
| Mean | 55% | 62% | 69% | 72% | 80% |
| Max | 69% | 81% | 86% | 86% | 92% |

| MediumKNN | | | | | |
|---|---|---|---|---|---|
| Channel | Gamma | Beta | alpha | Theta | Delta |
| Mean | 70% | 72% | 78% | 82% | 86% |
| Max | 75% | 83% | 86% | 89% | 92% |

| LinearSVM | | | | | |
|---|---|---|---|---|---|
| Channel | Gamma | Beta | alpha | Theta | Delta |
| Mean | 67% | 69% | 76% | 79% | 85% |
| Max | 78% | 83% | 83% | 92% | 89% |

| QuadraticSVM | | | | | |
|---|---|---|---|---|---|
| Channel | Gamma | Beta | alpha | Theta | Delta |
| Mean | 59% | 66% | 75% | 79% | 84% |
| Max | 75% | 83% | 86% | 89% | 94% |

| CubicSVM | | | | | |
|---|---|---|---|---|---|
| Channel | Gamma | Beta | alpha | Theta | Delta |
| Mean | 58% | 66% | 70% | 76% | 81% |
| Max | 75% | 81% | 83% | 92% | 94% |

| FineGuassianSVM | | | | | |
|---|---|---|---|---|---|
| Channel | Gamma | Beta | alpha | Theta | Delta |
| Mean | 69% | 70% | 70% | 72% | 70% |
| Max | 78% | 75% | 78% | 81% | 75% |

| CoarseKNN | | | | | |
|---|---|---|---|---|---|
| Channel | Gamma | Beta | alpha | Theta | Delta |
| Mean | 69% | 69% | 69% | 69% | 69% |
| Max | 69% | 69% | 69% | 69% | 69% |

| CosineKNN | | | | | |
|---|---|---|---|---|---|
| Channel | Gamma | Beta | alpha | Theta | Delta |
| Mean | 70% | 73% | 80% | 82% | 85% |
| Max | 75% | 89% | 89% | 89% | 92% |

| CubicKNN | | | | | |
|---|---|---|---|---|---|
| Channel | Gamma | Beta | alpha | Theta | Delta |
| Mean | 69% | 72% | 79% | 81% | 86% |
| Max | 78% | 86% | 86% | 89% | 92% |

| WeightedKNN | | | | | |
|---|---|---|---|---|---|
| Channel | Gamma | Beta | alpha | Theta | Delta |
| Mean | 64% | 68% | 77% | 81% | 85% |
| Max | 75% | 83% | 83% | 92% | 92% |

## Young bad vs old bad:

| | FineTree | | | | |
|---|---|---|---|---|---|
| Channel | Gamma | Beta | alpha | Theta | Delta |
| Mean | 62% | 65% | 69% | 72% | 76% |
| Max | 84% | 82% | 87% | 87% | 92% |

| | MediumTree | | | | |
|---|---|---|---|---|---|
| Channel | Gamma | Beta | alpha | Theta | Delta |
| Mean | 62% | 65% | 69% | 72% | 76% |
| Max | 84% | 82% | 87% | 87% | 92% |

| | CoarseTree | | | | |
|---|---|---|---|---|---|
| Channel | Gamma | Beta | alpha | Theta | Delta |
| Mean | 62% | 65% | 68% | 73% | 77% |
| Max | 84% | 82% | 87% | 87% | 92% |

| | LogisticRegression | | | | |
|---|---|---|---|---|---|
| Channel | Gamma | Beta | alpha | Theta | Delta |
| Mean | 63% | 66% | 68% | 69% | 74% |
| Max | 79% | 76% | 84% | 79% | 84% |

| | MediumGuassianSVM | | | | |
|---|---|---|---|---|---|
| Channel | Gamma | Beta | alpha | Theta | Delta |
| Mean | 65% | 66% | 72% | 72% | 82% |
| Max | 74% | 76% | 82% | 84% | 89% |

| | CoarseGuassianSVM | | | | |
|---|---|---|---|---|---|
| Channel | Gamma | Beta | alpha | Theta | Delta |
| Mean | 66% | 66% | 66% | 66% | 66% |
| Max | 66% | 68% | 68% | 66% | 68% |

| | FineKNN | | | | |
|---|---|---|---|---|---|
| Channel | Gamma | Beta | alpha | Theta | Delta |
| Mean | 53% | 57% | 67% | 70% | 78% |
| Max | 74% | 82% | 82% | 95% | 87% |

| | MediumKNN | | | | |
|---|---|---|---|---|---|
| Channel | Gamma | Beta | alpha | Theta | Delta |
| Mean | 67% | 71% | 76% | 77% | 83% |
| Max | 76% | 79% | 84% | 87% | 89% |

| | LinearSVM | | | | |
|---|---|---|---|---|---|
| Channel | Gamma | Beta | alpha | Theta | Delta |
| Mean | 67% | 69% | 74% | 75% | 82% |
| Max | 76% | 82% | 84% | 87% | 89% |

| | QuadraticSVM | | | | |
|---|---|---|---|---|---|
| Channel | Gamma | Beta | alpha | Theta | Delta |
| Mean | 59% | 62% | 68% | 71% | 81% |
| Max | 84% | 79% | 89% | 87% | 92% |

| | CubicSVM | | | | |
|---|---|---|---|---|---|
| Channel | Gamma | Beta | alpha | Theta | Delta |
| Mean | 56% | 58% | 69% | 73% | 79% |
| Max | 76% | 79% | 87% | 87% | 92% |

| | FineGuassianSVM | | | | |
|---|---|---|---|---|---|
| Channel | Gamma | Beta | alpha | Theta | Delta |
| Mean | 64% | 66% | 66% | 66% | 66% |
| Max | 74% | 71% | 74% | 74% | 74% |

| | CoarseKNN | | | | |
|---|---|---|---|---|---|
| Channel | Gamma | Beta | alpha | Theta | Delta |
| Mean | 66% | 66% | 66% | 66% | 66% |
| Max | 66% | 66% | 66% | 66% | 66% |

| | CosineKNN | | | | |
|---|---|---|---|---|---|
| Channel | Gamma | Beta | alpha | Theta | Delta |
| Mean | 68% | 72% | 77% | 78% | 83% |
| Max | 76% | 87% | 87% | 89% | 92% |

| | CubicKNN | | | | |
|---|---|---|---|---|---|
| Channel | Gamma | Beta | alpha | Theta | Delta |
| Mean | 67% | 71% | 76% | 76% | 82% |
| Max | 79% | 79% | 87% | 89% | 87% |

| | WeightedKNN | | | | |
|---|---|---|---|---|---|
| Channel | Gamma | Beta | alpha | Theta | Delta |
| Mean | 61% | 66% | 72% | 74% | 82% |
| Max | 79% | 76% | 84% | 89% | 89% |

## Young bad vs old good:

| | FineTree | | | | |
|---|---|---|---|---|---|
| Channel | Gamma | Beta | alpha | Theta | Delta |
| Mean | 53% | 60% | 73% | 76% | 72% |
| Max | 91% | 86% | 95% | 100% | 95% |

| | MediumTree | | | | |
|---|---|---|---|---|---|
| Channel | Gamma | Beta | alpha | Theta | Delta |
| Mean | 53% | 60% | 73% | 76% | 72% |
| Max | 91% | 86% | 95% | 100% | 95% |

| | CoarseTree | | | | |
|---|---|---|---|---|---|
| Channel | Gamma | Beta | alpha | Theta | Delta |
| Mean | 53% | 60% | 73% | 76% | 72% |
| Max | 91% | 86% | 95% | 100% | 95% |

| | LogisticRegression | | | | |
|---|---|---|---|---|---|
| Channel | Gamma | Beta | alpha | Theta | Delta |
| Mean | 63% | 63% | 64% | 66% | 70% |
| Max | 86% | 82% | 100% | 100% | 100% |

| | MediumGuassianSVM | | | | |
|---|---|---|---|---|---|
| Channel | Gamma | Beta | alpha | Theta | Delta |
| Mean | 57% | 58% | 69% | 78% | 78% |
| Max | 68% | 77% | 86% | 95% | 95% |

| | CoarseGuassianSVM | | | | |
|---|---|---|---|---|---|
| Channel | Gamma | Beta | alpha | Theta | Delta |
| Mean | 59% | 59% | 59% | 59% | 59% |
| Max | 59% | 59% | 59% | 59% | 59% |

| | FineKNN | | | | |
|---|---|---|---|---|---|
| Channel | Gamma | Beta | alpha | Theta | Delta |
| Mean | 55% | 57% | 68% | 68% | 78% |
| Max | 77% | 82% | 91% | 95% | 100% |

| | MediumKNN | | | | |
|---|---|---|---|---|---|
| Channel | Gamma | Beta | alpha | Theta | Delta |
| Mean | 60% | 62% | 75% | 79% | 80% |
| Max | 82% | 82% | 91% | 91% | 91% |

| | LinearSVM | | | | |
|---|---|---|---|---|---|
| Channel | Gamma | Beta | alpha | Theta | Delta |
| Mean | 58% | 61% | 69% | 79% | 78% |
| Max | 86% | 77% | 86% | 100% | 95% |

| | QuadraticSVM | | | | |
|---|---|---|---|---|---|
| Channel | Gamma | Beta | alpha | Theta | Delta |
| Mean | 58% | 60% | 66% | 75% | 79% |
| Max | 77% | 91% | 86% | 95% | 100% |

| | CubicSVM | | | | |
|---|---|---|---|---|---|
| Channel | Gamma | Beta | alpha | Theta | Delta |
| Mean | 58% | 59% | 67% | 72% | 78% |
| Max | 82% | 91% | 86% | 95% | 100% |

| | FineGuassianSVM | | | | |
|---|---|---|---|---|---|
| Channel | Gamma | Beta | alpha | Theta | Delta |
| Mean | 58% | 58% | 61% | 61% | 61% |
| Max | 73% | 68% | 68% | 77% | 86% |

| | CoarseKNN | | | | |
|---|---|---|---|---|---|
| Channel | Gamma | Beta | alpha | Theta | Delta |
| Mean | 60% | 62% | 75% | 79% | 80% |
| Max | 82% | 82% | 91% | 91% | 91% |

| | CosineKNN | | | | |
|---|---|---|---|---|---|
| Channel | Gamma | Beta | alpha | Theta | Delta |
| Mean | 59% | 59% | 77% | 83% | 78% |
| Max | 77% | 77% | 86% | 95% | 91% |

| | CubicKNN | | | | |
|---|---|---|---|---|---|
| Channel | Gamma | Beta | alpha | Theta | Delta |
| Mean | 59% | 62% | 75% | 77% | 80% |
| Max | 77% | 73% | 91% | 91% | 95% |

| | WeightedKNN | | | | |
|---|---|---|---|---|---|
| Canais | Gamma | Beta | alpha | Theta | Delta |
| Mean | 59% | 62% | 75% | 77% | 80% |
| Max | 77% | 73% | 91% | 91% | 95% |

## Young good vs old good:

FineTree

| Channel | Gamma | Beta | alpha | Theta | Delta |
|---|---|---|---|---|---|
| Mean | 58% | 57% | 66% | 67% | 70% |
| Max | 85% | 85% | 90% | 90% | 90% |

MediumTree

| Channel | Gamma | Beta | alpha | Theta | Delta |
|---|---|---|---|---|---|
| Mean | 58% | 57% | 66% | 67% | 70% |
| Max | 85% | 85% | 90% | 90% | 90% |

CoarseTree

| Channel | Gamma | Beta | alpha | Theta | Delta |
|---|---|---|---|---|---|
| Mean | 58% | 57% | 66% | 67% | 70% |
| Max | 85% | 85% | 90% | 90% | 90% |

LogisticRegression

| Channel | Gamma | Beta | alpha | Theta | Delta |
|---|---|---|---|---|---|
| Mean | 58% | 62% | 61% | 64% | 65% |
| Max | 90% | 85% | 85% | 85% | 95% |

MediumGuassianSVM

| Channel | Gamma | Beta | alpha | Theta | Delta |
|---|---|---|---|---|---|
| Mean | 58% | 53% | 68% | 71% | 76% |
| Max | 80% | 75% | 80% | 90% | 95% |

CoarseGuassianSVM

| Channel | Gamma | Beta | alpha | Theta | Delta |
|---|---|---|---|---|---|
| Mean | 55% | 55% | 55% | 55% | 55% |
| Max | 55% | 55% | 55% | 55% | 55% |

FineKNN

| Channel | Gamma | Beta | alpha | Theta | Delta |
|---|---|---|---|---|---|
| Mean | 50% | 56% | 59% | 63% | 72% |
| Max | 75% | 85% | 80% | 95% | 90% |

MediumKNN

| Channel | Gamma | Beta | alpha | Theta | Delta |
|---|---|---|---|---|---|
| Mean | 57% | 57% | 72% | 77% | 80% |
| Max | 75% | 70% | 85% | 85% | 90% |

LinearSVM ★

| Channel | Gamma | Beta | alpha | Theta | Delta |
|---|---|---|---|---|---|
| Mean | 59% | 56% | 67% | 70% | 77% |
| Max | 90% | 70% | 90% | 90% | 95% |

QuadraticSVM

| Channel | Gamma | Beta | alpha | Theta | Delta |
|---|---|---|---|---|---|
| Mean | 62% | 60% | 63% | 69% | 75% |
| Max | 90% | 85% | 85% | 90% | 95% |

CubicSVM

| Channel | Gamma | Beta | alpha | Theta | Delta |
|---|---|---|---|---|---|
| Mean | 59% | 60% | 63% | 70% | 72% |
| Max | 85% | 85% | 90% | 85% | 90% |

FineGuassianSVM

| Channel | Gamma | Beta | alpha | Theta | Delta |
|---|---|---|---|---|---|
| Mean | 52% | 50% | 54% | 55% | 60% |
| Max | 65% | 65% | 75% | 75% | 90% |

CoarseKNN

| Channel | Gamma | Beta | alpha | Theta | Delta |
|---|---|---|---|---|---|
| Mean | 55% | 55% | 55% | 55% | 55% |
| Max | 55% | 55% | 55% | 55% | 55% |

CosineKNN

| Channel | Gamma | Beta | alpha | Theta | Delta |
|---|---|---|---|---|---|
| Mean | 55% | 56% | 70% | 77% | 78% |
| Max | 75% | 75% | 80% | 85% | 90% |

CubicKNN

| Channel | Gamma | Beta | alpha | Theta | Delta |
|---|---|---|---|---|---|
| Mean | 58% | 56% | 68% | 73% | 80% |
| Max | 75% | 80% | 80% | 90% | 90% |

WeightedKNN

| Channel | Gamma | Beta | alpha | Theta | Delta |
|---|---|---|---|---|---|
| Mean | 56% | 52% | 64% | 73% | 77% |
| Max | 75% | 75% | 85% | 85% | 85% |

## Old good vs old bad:

FineTree

| Channel | Gamma | Beta | alpha | Theta | Delta |
|---|---|---|---|---|---|
| Mean | 61% | 61% | 60% | 63% | 67% |
| Max | 85% | 79% | 85% | 85% | 85% |

MediumTree

| Channel | Gamma | Beta | alpha | Theta | Delta |
|---|---|---|---|---|---|
| Mean | 61% | 61% | 60% | 63% | 67% |
| Max | 85% | 79% | 85% | 85% | 85% |

CoarseTree

| Channel | Gamma | Beta | alpha | Theta | Delta |
|---|---|---|---|---|---|
| Mean | 61% | 61% | 60% | 63% | 68% |
| Max | 85% | 79% | 85% | 85% | 85% |

LogisticRegression

| Channel | Gamma | Beta | alpha | Theta | Delta |
|---|---|---|---|---|---|
| Mean | 64% | 63% | 58% | 61% | 65% |
| Max | 71% | 71% | 74% | 71% | 82% |

MediumGuassianSVM

| Channel | Gamma | Beta | alpha | Theta | Delta |
|---|---|---|---|---|---|
| Mean | 74% | 74% | 73% | 73% | 73% |
| Max | 79% | 79% | 74% | 74% | 74% |

CoarseGuassianSVM

| Channel | Gamma | Beta | alpha | Theta | Delta |
|---|---|---|---|---|---|
| Mean | 74% | 74% | 74% | 74% | 74% |
| Max | 74% | 74% | 74% | 74% | 74% |

FineKNN

| Channel | Gamma | Beta | alpha | Theta | Delta |
|---|---|---|---|---|---|
| Mean | 64% | 63% | 59% | 59% | 62% |
| Max | 82% | 82% | 74% | 76% | 79% |

MediumKNN

| Channel | Gamma | Beta | alpha | Theta | Delta |
|---|---|---|---|---|---|
| Mean | 73% | 74% | 73% | 74% | 74% |
| Max | 76% | 76% | 76% | 76% | 79% |

LinearSVM ★

| Channel | Gamma | Beta | alpha | Theta | Delta |
|---|---|---|---|---|---|
| Mean | 72% | 72% | 72% | 71% | 71% |
| Max | 85% | 82% | 74% | 76% | 79% |

QuadraticSVM

| Channel | Gamma | Beta | alpha | Theta | Delta |
|---|---|---|---|---|---|
| Mean | 68% | 67% | 63% | 65% | 67% |
| Max | 85% | 85% | 76% | 79% | 85% |

CubicSVM

| Channel | Gamma | Beta | alpha | Theta | Delta |
|---|---|---|---|---|---|
| Mean | 64% | 67% | 60% | 59% | 65% |
| Max | 85% | 88% | 74% | 79% | 79% |

FineGuassianSVM

| Channel | Gamma | Beta | alpha | Theta | Delta |
|---|---|---|---|---|---|
| Mean | 74% | 74% | 73% | 73% | 73% |
| Max | 79% | 79% | 74% | 76% | 74% |

CoarseKNN

| Channel | Gamma | Beta | alpha | Theta | Delta |
|---|---|---|---|---|---|
| Mean | 74% | 74% | 74% | 74% | 74% |
| Max | 74% | 74% | 74% | 74% | 74% |

CosineKNN

| Channel | Gamma | Beta | alpha | Theta | Delta |
|---|---|---|---|---|---|
| Mean | 74% | 73% | 74% | 74% | 74% |
| Max | 76% | 74% | 76% | 76% | 79% |

CubicKNN

| Channel | Gamma | Beta | alpha | Theta | Delta |
|---|---|---|---|---|---|
| Mean | 74% | 74% | 73% | 73% | 74% |
| Max | 74% | 74% | 76% | 74% | 79% |

WeightedKNN

| Channel | Gamma | Beta | alpha | Theta | Delta |
|---|---|---|---|---|---|
| Mean | 74% | 74% | 73% | 73% | 74% |
| Max | 74% | 74% | 76% | 74% | 79% |

## Young good vs young bad:

| | | | | | |
|---|---|---|---|---|---|
| | FineTree | | | | |
| Channel | Gamma | Beta | alpha | Theta | Delta |
| Mean | 47% | 48% | 49% | 53% | 49% |
| Max | 71% | 75% | 71% | 83% | 79% |

| | | | | | |
|---|---|---|---|---|---|
| | MediumTree | | | | |
| Channel | Gamma | Beta | alpha | Theta | Delta |
| Mean | 47% | 48% | 49% | 53% | 49% |
| Max | 71% | 75% | 71% | 83% | 79% |

| | | | | | |
|---|---|---|---|---|---|
| | CoarseTree | | | | |
| Channel | Gamma | Beta | alpha | Theta | Delta |
| Mean | 47% | 48% | 49% | 53% | 49% |
| Max | 71% | 75% | 71% | 83% | 79% |

| | | | | | |
|---|---|---|---|---|---|
| | LogisticRegression | | | | |
| Channel | Gamma | Beta | alpha | Theta | Delta |
| Mean | 43% | 50% | 49% | 47% | 50% |
| Max | 63% | 75% | 88% | 71% | 75% |

| | | | | | |
|---|---|---|---|---|---|
| | MediumGuassianSVM | | | | |
| Channel | Gamma | Beta | alpha | Theta | Delta |
| Mean | 50% | 52% | 47% | 46% | 48% |
| Max | 63% | 79% | 63% | 67% | 58% |

| | | | | | |
|---|---|---|---|---|---|
| | CoarseGuassianSVM | | | | |
| Channel | Gamma | Beta | alpha | Theta | Delta |
| Mean | 54% | 54% | 54% | 54% | 54% |
| Max | 54% | 54% | 54% | 54% | 54% |

| | | | | | |
|---|---|---|---|---|---|
| | FineKNN | | | | |
| Canais | Gamma | Beta | alpha | Theta | Delta |
| Média | 43% | 48% | 49% | 47% | 55% |
| Maximo | 67% | 88% | 79% | 71% | 75% |

| | | | | | |
|---|---|---|---|---|---|
| | MediumKNN | | | | |
| Channel | Gamma | Beta | alpha | Theta | Delta |
| Mean | 57% | 61% | 56% | 56% | 59% |
| Max | 75% | 75% | 75% | 75% | 71% |

| | | | | | |
|---|---|---|---|---|---|
| | LinearSVM | | | | |
| Channel | Gamma | Beta | alpha | Theta | Delta |
| Mean | 49% | 53% | 45% | 44% | 47% |
| Max | 67% | 75% | 71% | 75% | 79% |

| | | | | | |
|---|---|---|---|---|---|
| | QuadraticSVM | | | | |
| Channel | Gamma | Beta | alpha | Theta | Delta |
| Mean | 43% | 48% | 49% | 52% | 56% |
| Max | 71% | 79% | 75% | 67% | 75% |

| | | | | | |
|---|---|---|---|---|---|
| | CubicSVM | | | | |
| Canais | Gamma | Beta | alpha | Theta | Delta |
| Média | 46% | 51% | 49% | 53% | 55% |
| Maximo | 67% | 79% | 71% | 71% | 71% |

| | | | | | |
|---|---|---|---|---|---|
| | FineGuassianSVM | | | | |
| Channel | Gamma | Beta | alpha | Theta | Delta |
| Mean | 48% | 51% | 51% | 51% | 51% |
| Max | 71% | 71% | 75% | 71% | 67% |

| | | | | | |
|---|---|---|---|---|---|
| | CoarseKNN | | | | |
| Channel | Gamma | Beta | alpha | Theta | Delta |
| Mean | 54% | 54% | 54% | 54% | 54% |
| Max | 54% | 54% | 54% | 54% | 54% |

| | | | | | |
|---|---|---|---|---|---|
| | CosineKNN | | | | |
| Channel | Gamma | Beta | alpha | Theta | Delta |
| Mean | 60% | 58% | 56% | 54% | 54% |
| Max | 83% | 79% | 71% | 71% | 79% |

| | | | | | |
|---|---|---|---|---|---|
| | CubicKNN | | | | |
| Channel | Gamma | Beta | alpha | Theta | Delta |
| Mean | 58% | 61% | 56% | 56% | 61% |
| Max | 75% | 75% | 75% | 71% | 75% |

| | | | | | |
|---|---|---|---|---|---|
| | WeightedKNN | | | | |
| Channel | Gamma | Beta | alpha | Theta | Delta |
| Mean | 47% | 47% | 46% | 47% | 51% |
| Max | 67% | 75% | 63% | 71% | 67% |

**Regions that presented higher differences**

| **EEG brain configuration changes in theta and delta subbands in young vs older adults** | | | | |
|---|---|---|---|---|
| **Subband** | **YG vs OB** | **YB vs OG** | **YB vs OB** | **YG vs OG** |
| Theta | D4 | C4 | B26 | D19 |
| | B2 | **B16** | **A30** | |
| | **A30** | A19 | | |
| Delta | **B16** | C7 | **B16** | D26 |
| | A23 | A23 | | |
| | | A19 | | |
| | | A15 | | |

**Note.** Table 2 displays the channels that presented higher differences between the study groups in the theta and delta subbands. YG = Young Adults with Good Sleep Quality; OB = Older Adults with Bad sleep Quality; YB = Young Adults Bad Sleep Quality; OG = Older Adults Good sleep Quality; Highlighted *in blue* the most representative channels indicating bad sleep in ageing; Channels in *in green* indicate age related changes; *In orange* the channel related to bad sleep independently of age. Only the theta and delta subbands are displayed as they are the ones that showed greater accuracy levels. (See below a biosemi plot with the biosemi EEG channels' locations). In *black* changes due to age and / or sleep (difficult to disentangle).

| **Regions most affected when comparing the older groups** | |
|---|---|
| **Subband** | **OG vs OB** |
| Gamma | A23 |
| Beta | A23 |
| Alpha | Several |
| Theta | A1 |
| Delta | C29 |
| | C7 |

**Note.** Table 3 displays the channels that presented higher differences between the study groups. OG = Older with Good Sleep Quality and the OB =Older with Bad Sleep Quality. In this table, all the subbands are displayed as accuracy levels are homogeneous across them.
